# Histochemical and Immunohistochemical Analysis of Collagen Fibers and Microvascular Density in Various Grades of Oral Submucous Fibrosis

**DOI:** 10.30699/IJP.14.2.127

**Published:** 2019-01-10

**Authors:** Savita S Thakkannavar, Veena V Naik

**Affiliations:** 1 *Lecturer, Department of Oral Pathology & Microbiology, Tatyasaheb Kore Dental College & Research Centre, New paragon, Kolhapur, India*; 2 *Professor, Department of Oral Pathology & Microbiology, KLE University, KLE VK Institute of Dental Sciences, Belgaum, India*

**Keywords:** Immunohistochemistry, Morphometry, Oral Submucous Fibrosis, Picrosirus red, Muscle Fibers

## Abstract

**Background and Objective:**

This study was aimed to evaluate the collagen fibers qualitatively and its correlation with microvascular density in various grades of oral submucous fibrosis (OSMF).

**Material and Methods:**

The present study comprised of total 40 cases of oral submucous fibrosis. Picrosirius red staining was done on all the specimens’ sections. They were analyzed for the colour and orientation of collagen fibers. Morphometric measurements were done using image analysis on immunohistochemical stained sections for Factor VIII-related antigen and analyzed for microvascular density.

**Results:**

Picrosirius red polarizing microscopy results revealed that there was a shift in the colour of collagen fibers from greenish yellow to orange red and red colour as the severity of the oral submucous fibrosis increased. The collagen fibers showed mixed orientation in early oral submucous fibrosis and parallel orientation in advanced oral submucous fibrosis. There was a significant decrease in microvascular density from early to advanced oral submucous fibrosis.

**Conclusion:**

The change in the colours and orientation of collagen fibers in early and advanced oral submucous fibrosis could be attributed to the fibre thickness, type of collagen, alignment and packing, cross-linking of the fibers and the section thickness. However, in advanced cases the vascularity is reduced which may predispose to epithelial atrophy and subsequent malignant changes.

## Introduction

 Oral submucous fibrosis (OSMF) is a chronic insidious disease, predominantly affecting the people with South-East Asian origin (1). It is measured as a premalignant condition of oral cancer, and the malignant transformation risk varies from 2.3 to 7.6%. Once the disease has settled, there is neither regression nor any effective treatment (2). Current evidence advocate that alkaloids namely arecoline, arecadine and tannins present in the arecanut are the key factors which initiate the disease process (3). It is apparent that the fibrosis and hyalinization of the sub-epithelial tissues account for the most of the clinical features which encounter in OSMF. Hence, it is rational to speculate that the increase in collagen synthesis or reduction in collagen degradation as possible mechanism in the development of the disease process and the normal regulatory mechanisms are either up-regulated or down-regulated at different stages of the OSMF (4). Collagen is a fundamental part of the connective tissue in every organ in the body. The study of collagen has emphasised the knowledge of collagen biosynthesis and degradation, which is a complex mechanism. Since collagen makeup nearly 30% of all the proteins in the body, it is essential to use available research tools to study collagen in the health issue as well as disease (5). Numerous forms of special stains have been used to detect collagen fibers but the picrosirius red stain demonstrated superior results compared to the other special stains. The collagen fibers were stained intensely with spectacular birefringence. These stains are more stable and do not fade easily (6). Angiogenesis is a multistep, highly orchestrated process which is involved in sprouting of vessel, endothelial cell migration, proliferation, tube formation and survival. In most of the studies, as a measurement of angiogenic activity, the number of microvessels in tissue sections is counted and expressed as microvascular density (MVD) (7). There is a significant increase in vascularity during the transition from normal oral mucosa through various degrees of dysplasia, and invasive squamous cell carcinoma (8). Immunohistochemical staining of microvessels using markers like VEGF, CD31 and Factor VIII-related antigen to assess MVD per unit area is associated with the degree of intratumor neovascularisation, tumor metastatic capability and the prognosis for patients with many types of human solid cancers (9). Von Willebrand factor (vWF) is a blood glycoprotein which is required for the normal haemostasis. The vWF mediates the adhesion of platelets to the sites of vascular damage by binding to specific platelet membrane glycoproteins and to constituents of the exposed connective tissue. Factor VIII-related antigen (RAG) has been used widely as a marker for endothelial cells in numerous immunohistological studies (10). Morphometry is a quantitative basic system which empowers target objective information to be gained from structures of the cells and tissues. The upside of morphometric examination is its objectivity and reproducibility which empowers guide correlations from individual to individual and centre to centre (11). As there is scarcity of information in the literature correlating collagen fibers and angiogenic activity in OSMF, the present study was aimed to evaluate the collagen fibers in various grades of OSMF and correlate it with MVD. 

## Materials and Methods

Following approval from the KLE VK Institute of Dental Sciences, Belgaum, a total of 40 formalin-fixed paraffin-embedded (FFPE) tissue blocks of oral submucous fibrosis with various grades were retrieved from the Department of Oral Pathology and Microbiology. Tissue blocks belonged to 40 patients of which 36 were males and 4 were females. The patients referred with clinically palpable fibrous bands, mild to severe burning sensation of oral cavity with reduction in mouth opening. The OSMF group was subdivided into very early, early, moderately advanced and advanced OMSF according to the Pindborg and Sirsat criteria (12).

**Very early stage: **Finely fibrillar collagen dispersed with marked edema; Plump young fibroblast containing abundant cytoplasm; Dilated and congested blood vessels; Finding inflammatory cells, mainly polymorphonuclear leukocytes with occasional eosinophils. 

**Early stage:** Juxta-epithelial area which shows early hyalinization; Collagen still in separate thick bundles; The presence of moderate number of plump young fibroblasts. Dilated and congested blood vessels. Inflammatory cells which are primarily lymphocytes, eosinophils and occasional plasma cells. 

**Moderately advanced stage:** Moderately hyalinized collagen; Thickened collagen bundles separated by slight residual edema; Less marked fibroblastic response; Either normal or compressed blood vessels; Inflammatory exudate consists of lymphocytes and plasma cells. 

**Advanced stage:** Completely hyalinized collagen; A smooth sheet with no separate bundles of collagen; Absence of edema; Hyalinized area devoid of fibroblasts; Completely obliterated or narrowed blood vessels; Lymphocytes and plasma cells as inflammatory cells^.^ (12^TM)^ picture analysis programming (version 3.5.0) with research microscope (Leica^TM^ DM2500). To determine the MVD as specified by Weidner et al., any brown stained endothelial cell which was clearly separated from adjacent microvessels, tumor cells, and other connective tissue cells elements was considered to be a single countable microvessel. The picture of five agent fields in the subepithelial district from each segment were caught in a stepwise manner by moving the microscope stage from left to right utilizing CCD shading camcorder (Leica DFC 320) joined to the microscope. The pictures were envisioned and stored in a computer for advance investigation (11).

Blood vessels were outlined with a cursor at a magnification of X400. The blood vessels were measured for mean vascular density. The mean average scores were tabulated and used for the statistical analysis by Fischer’s exact test. 

## Results

**Figure 1:**
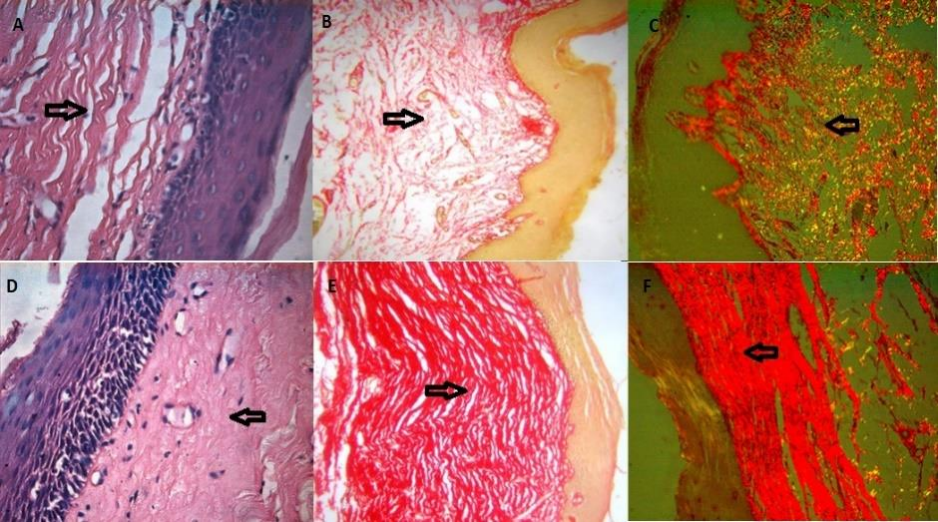
H & E stained sections of EOSMF: (A; X400). Picrosirus red stain showing mixed orientation of the collagen fibers: (B; X400). Picrosirus red stain under polarizing microscopy showing greenish color with mixed orientation of collagen fibers: (C; X400). H & E stained section of AOSMF: (D; X400). Picrosirus red stain showing parallel orientation of collagen fibers: (E; X400). Picrosirus red stain under polarizing microscopy showing red color with parallel orientation of collagen fibers: (F; X400)

The picrosirius red stained sections were assessed for the colour and orientation under polarizing microscopy (Figure 1).

**Figure 2:**
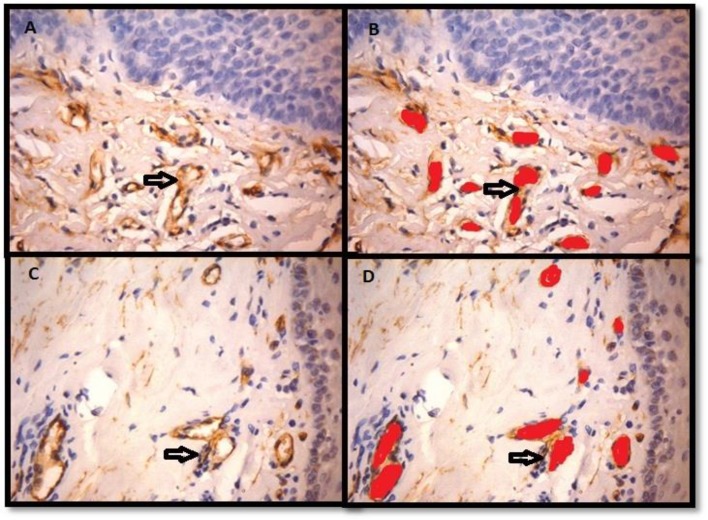
It shows Factor VIII-related antigen immunostained section of EOSMF (A; X400) and AOSMF (C; X400). Blood vessels marked for morphometric analysis in the sub-epithelial region of EOSMF (B; X400) and AOSMF (D; X400)

The collagen fibers in 70% of EOSMF showed greenish yellow (GY) colour, 10% showed orange red (OR) and 10% showed yellowish orange (YO) colour, whereas the collagen fibers in 60% cases of AOSMF showed orange red colour (OR) and 30% showed red (R) colour (Table 1). Ten cases of EOSMF showed mixed orientation of collagen fibers, 7 and 3 cases showed non-parallel and parallel orientations, respectively. In AOSMF, 70% of cases showed parallel orientation of collagen fibers whereas 30% of cases showed mixed orientation and none showed non-parallel orientation of collagen fibers (Table 2). Immunohistochemical staining with Factor VIII-related antigen was undertaken in all the cases followed by morphometric analysis of the vasculature for microvascular density (Figure 2).

**Table 1:** Polarizing colour variation of collagen fibers in early and advanced oral submucous fibrosis

Group	Colour						Total
	GY	YO	OR	R	YR	GO	
Early OSMF	14(70.0%)	2(10.0%)	2(10.0%)	1(5.0%)	0(0.0%)	1(5.0%)	20
Advanced OSMF	0(0.0%)	0(0.0%)	12(60.0%)	6(30.0%)	1(5.0%)	1(5.0%)	20
Total	14(35.0%)	2(5.0%)	14(35.0%)	7(17.5%)	1(2.5%)	2(5.0%)	40
*P-value*	0.00*						

GY: Greenish Yellow, YO: Yellowish orange, OR: Orange Red, R: Red, YR: Yellowish Red, GO: Greenish Orange

**Table 2:** Orientation of collagen fibers in early and advanced oral submucous fibrosis

Group	Orientation			Total
	Mixed	Non-parallel	Parallel	
Early OSMF	10(50.0%)	7(35.0%)	3(15.0%)	20
Advanced OSMF	6(30.0%)	0(0.0%)	14(70.0%)	20
Total	16(40.0%)	7(7.5%)	17(42.5%)	40(100.0%)
*P-value*	0.00*			

* Fisher’s exact test, *P-value* < 0.05: statistically significant

We counted 5 microscopic fields with X400 magnification with one field depth from the basement membrane of the epithelium. The mean of microvascular density (MVD) of blood vessels were analyzed in both groups and the values were calculated. The MVD ranged from 6.4 to 13.4 in all OSMF cases with the mean MVD in EOSMF and AOSMF found to be 9.9 ± 1.61 and 7.65 ± 1.22, respectively. There was significant decrease in MVD from EOSMF to AOSMF (Table 3). The correlation between MVD and collagen fibers was not significant in EOSMF as well as AOSMF (Table 4). 

**Table 3:** Microvascular density in Early and Advanced oral submucous fibrosis

Group	MVD	Standard deviation	t	P-value
Early OSMF	9.9	1.61	4.95	0.00*
Advanced OSMF	7.65	1.22	4.95	0.00*

* P-value < 0.05: statistically significant

**Table 4:** Correlation between MVD and collagen fibres in EOSMF and AOSMF

	EOSMF		AOSMF	
	Colour	Orientation	Colour	Orientation
MVD	r =0.31 *P*= 0.896	r =-0.239 *P*= 0.311	r =-0.52 *P*= 0.828	r = -0.219 *P*= 0.354

## Discussion

Oral submucous fibrosis is characterized by inflammation and progressive mucosal fibrosis. In early stages it shows epithelial hyperplasia and atrophy in later stages. The changes in connective tissue vary from fibrosis to hyalinization (12). Picrosirius red stain has consistently provided excellent results for the demonstration of collagen fibers. This is due to the parallel relationship between the collagen fibers and the dye molecules which results in an enhanced birefringence. Even the delicate collagen fibers which are undetectable under normal microscope become visible by polarizing light microscopy (7, 16). The collagen molecules are rich in basic amino acids that react strongly with acidic dyes. In histological sections, the Sirius red dye is used at 0.1% concentration in a saturated picric acid solution to prevent indiscriminate staining of non-collagenous structures. The light intensity is increased due to birefringence of collagen. Poorly formed collagen fibers are shown greenish whereas, in a more mature stage, became yellow, orange or red under crossed. Thus, Picrosirius polarization light technique could be useful to form an opinion about the activity of the fibrotic process (13). Atrophic changes in the epithelium are accepted to be auxiliary to the connective tissue changes. The level of vascularity of the ailing mucosa and its part on the epithelial thickness in OSMF has been dependably involved in the verbal confrontation (14).

Image analysis forms an important tool in quantification of histopathology tests’ results. It is used to extract information contained in images for detection, diagnosis and description of various features. This method has been used for the quantification of vasculature in pre-malignant and malignant conditions. Currently, the image analysis system has allowed improving the potential accuracy of morphometric studies. Despite the fact that angiogenesis can't be measured straightforwardly, it can be induced by evaluation of the vasculature, more often as MVD, therefore giving a file of angiogenesis. Microvascular density is the mean estimation of microvessel count, acquired utilizing a particular target amplification with known field distance across a selected microscope in a set number of fields, subjectively chosen from the vascularised territories (Hot spots) (14).

The generally acceptable criteria for a microvessel profile are endothelial microvessel profiles present within the tumor but not in the necrotic or sclerotic zones. The present results suggest that majority of the collagen fibers in early OSMF showed green to greenish yellow colour and there was a shift from orange red to red colour with increased severity of OSMF. It is similar to a study conducted by Ceena DE et al., suggesting that tight packing of collagen fibers in OSMF is progressively increased as the disease progresses from early to advanced stage (15). The birefringent colours of collagen fibers are ranged from green and greenish yellow to orange and red. The differences in the collagen birefringence are attributed to the fiber thickness, type of collagen, alignment and packing, cross-linking of the fibers and the section thickness. The polarization colours were determined not only by the fiber thickness but also by packing of the collagen molecules. The tightly packed and presumably, better aligned were of longer wavelength and poorly packed fibers were of shorter wavelength with green to yellow birefringence. These findings were confirmed by Sharf Y et al., in their Double Quantum-Filtered NMR Spectroscopy study (16). According to a study conducted on the collagen fibers, majority of the collagen fibers were oriented parallel to the epithelium, with a statistically significant difference in orientation between oral submucous fibrosis and control groups. The purpose behind uni-directional arrangement of clinical fibrous bands could be because of endless incitement of oral mucosa by the irritants prompting changes in the orientation of the collagen fiber bundles which may bring about scar development like that of wound healing, where the collagen strands are situated parallel to the epidermis (17,18,19). In the present study the morphometric analysis of the blood vessels was carried out for microvascular density on immunostained specimens with Factor VIII-related antigen. This was in concurrence with the previous studies by Desai et al who found that MVD increased in the early stages and decreased in the advanced stages and concluded that it could be due to the presence of microvessel hyperplasia that occurred in early stage of OSMF (14). In contrast, the study by Sabarinath et al., showed that MVD increased with advancing stages of OSMF, and it was statistically insignificant (7). A study conducted on morphometric analysis of OSMF found that blood vessel density, mean blood vessel area and mean diameter of the vessels were indirectly proportional to the histological stages of OSMF (20). Histological stages directly correlate with the frequency of trismus, but the severity of trismus showed relation neither with the staging nor with the degree of collagenization, measured morphometrically. The mean blood vessel area and the mean vessel diameter showed a noticeable increase in the early stage. The luminal diameter in the advanced stage showed near obliteration of the lumen (21). In contrast to our study, the image analysis for the quantitative assessment of the mucosal vasculature in OSMF showed that mean vascular density was more or less the same in the test and control samples, which could be due to the absence of stromal preconditioning which is required to induce neo-angiogenesis (20). In our study, the assessment of the correlation between MVD and collagen fibers was not found to be significant. The differences of MVD in EOSMF and AOSMF were statistically significant (*P <*0.05). The rise in MVD noted in the early stages of submucous fibrosis is probably for the mucosa attempts to cope with the hypoxia created due to fibrosis by actively promoting neoangiogenesis. As the disease progresses, the stroma gets more hyalinized, the mediators of angiogenesis seems to diminish in concentration which could be the reason for the decrement in MVD observed in advanced stage of OSMF (22). The study showed statistical significant difference in colour, orientation and MVD between EOSMF and AOSMF (*P<0.05*). The majority of the collagen fibers in EOSMF showed greenish yellow colour whereas in AOSMF the majority of the collagen fibers showed orange red to red colour which could be contributed to the alignment and packing of the collagen fibers. 

## Conclusion

The changes in the colours and orientation of the collagen fibers in early and advanced oral submucous fibrosis could be attributed to the fiber thickness, type of collagen, alignment and packing, cross-linking of the fibers and the section thickness. Decrease in the vascularity from early to advanced OMSF may predispose to atrophic changes in overlying epithelium and subsequent malignant changes.
